# Goal-directed therapy in cardiovascular surgery: A case series study

**DOI:** 10.34172/jcvtr.2023.31838

**Published:** 2023-09-23

**Authors:** Nader Givtaj, Elnaz Hosseinzadeh, Fatemeh Shima Hadipourzadeh, Zahra Faritous, Mohammad Hasan Askari, Maryam Ghanbari Garekani

**Affiliations:** Rajaie Cardiovascular Medical and Research Center, Iran University of Medical Sciences, Tehran, Iran

**Keywords:** Advanced hemodynamic monitoring, Cardiovascular surgery, Goal directed therapy

## Abstract

Hemodynamic and intravascular volume monitoring has been utilized and significantly improved thanks to the technology revolution. Goal-Directed Therapy (GDT) derived from this advanced monitoring is beneficial for complex surgeries, and it shifted the medical approaches from static therapy to more personalized functional treatments. Conventional monitoring methods such as blood pressure, heart rate, urinary output, and central venous pressure are commonly used. However, studies have shown these routine parameters often cannot precisely estimate the quality of tissue perfusion. Tissue hypoperfusion and hypoxia play a crucial role in initiating a systemic inflammatory response after prolonged surgeries, resulting in unstable hemodynamic condition of the patients. Several studies reported the importance of GDT in non-cardiac surgeries and there are few reports on cardiac surgeries. However, tissue perfusion and fluid management are more critical in complex and prolonged cardiovascular surgeries to avoid complications such as low cardiac output syndrome and renal or pulmonary dysfunction. Different advanced hemodynamic monitorings have been utilized perioperatively in cardiac surgery to help decision-making on inotrope and fluid management. In this article we present 5 cases of usefulness hemodynamic monitoring in patients who underwent cardiovascular surgeries.

## Introduction

 Goal Directed Therapy (GDT) in cardiac surgery is the use of various hemodynamic parameters (such as stroke volume, cardiac output, systemic vascular resistance and stroke volume variation) and optimizing fluids, inotropes and vasopressors to ensure sufficient tissue perfusion^1,2^ (Table 1). The optimization of the oxygen delivery rate by these advanced hemodynamic parameters was investigated for the first time in the 1980s by Shoemaker et al.^3,4^ They observed that in patients who survived cardiogenic shock, the cardiac index, oxygen transport, and oxygen consumption were significantly higher than other patients who were lost due to shock. Their study in 1988 showed that, high risk and complicated patients who subjected GDT had lower mortality and hospital stay. Others also reported similar results, however, the debate over the performance of GDT continued.^5,6^

**Table 1:** Overview of the techniques and hemodynamic parameters of the advanced hemodynamic monitoring platforms used in this study

**Sensor/Catheter**	**Compatible Platform**	**Technique**	**Invasiveness**	**Advanced hemodynamic parameters**
FloTrac	Vigileo EV1000 HemoSphere	Arterial pulse pressure algorithm, updating every 20 second, uncalibrated	Minimal Invasive	SV, SVV, MAP, SVR, CO
Swan-Ganz	Vigilance l & ll HemoSphere	Pulmonary artery catheter	Invasive	SV, CO, SVR, RVEF, RVEDV, PAP, PAOP
Swan-Ganz CCOmbo	Vigilance l & ll -HemoSphere	Pulmonary artery catheter with oximetry (mixed venous oxygen saturation (SvO_2_) updating every 2 seconds)	Invasive	SV, CO, SVR, RVEF, RVEDV, PAP, PAOP + SvO_2_
Volume View	EV1000	Transpulmonary Thermodilution (TPTD), with two catheters for femoral artery & central venous – Calibrated	Less Invasive	CO, CI, SV, SVR, SVRI & Volumetric parameters: EVLW, PVPI, GEDV and GEF
PediaSat	Vigileo HemoSphere	Pediatric oximetry catheter, Real-time monitoring of central venous oxygen saturation (ScvO_2_)	Less Invasive	ScvO_2_
Central Venous Oximetry	Vigileo	Adult oximetry catheter, Real-time monitoring of central venous oxygen saturation (ScvO_2_)	Less Invasive	ScvO_2_

Stroke Volume (SV), Stroke Volume Variation (SVV), Mean Arterial Pressure (MAP), Systemic Vascular Resistance (SVR), Systemic Vascular Resistance Index (SVRI), Cardiac Output (CO), Cardiac Index (CI), Right ventricular ejection fraction (RVEF), Right ventricular end diastolic volume (RVEDV), Pulmonary artery pressure (PAP), Pulmonary artery occlusion pressure (PAOP), Extravascular Lung Water (EVLW), Pulmonary Vascular Permeability Index (PVPI), Global End-Diastolic Volume (GEDV), Global ejection fraction (GEF).

 The purpose of GDT is to use flow-based hemodynamic parameters that can manage the amount of fluid, inotropes, medication and tissue blood flow and oxygenation. In other terms, normal tissue perfusion prevents inflammatory responses and possible fatal complications which reduces the mortality rate.^7,8^

 GDT has been extensively investigated in several clinical trial studies and has provided favorable results in complex non-cardiac surgeries.^9^ However, few reports are available regarding the role of GDT in complex cardiac procedures. Hence, we present 5 cases of cardiovascular surgery in which advanced hemodynamic monitoring played significant role in management of patients.

## Case Presentations

###  Case 1

 A 53-year-old man with a history of diabetes, hypertension, and addiction was a candidate for pulmonary endarterectomy due to Chronic Thromboembolic Pulmonary Hypertension (CTEPH). The pre-operative echocardiography showed an ejection fraction of 55%, moderate to severe right ventricular dysfunction, severe right atrial enlargement, mild to moderate mitral regurgitation, systolic pulmonary pressure of 77 mm Hg, tricuspid regurgitation with gradient of 62 mm Hg, and severe pulmonary hypertension. In addition to routine monitoring for cardiac surgery, Pulmonary Artery Catheter, Swan–Ganz catheter (SGC) (Edwards Lifesciences, Irvine, CA, USA) and Volume View^TM^/EV1000^TM^ (Edwards Lifesciences, Irvine, CA, USA) were used.^10,11^ The surgery was uneventful and the patient was weaned from cardiopulmonary bypass with the support of epinephrine (0.1mcg/kg/min), milrinone (0.5 mcg/kg/min) and vasopressin (2u/h) and pulmonary artery pressure dropped from 77/21 mm Hg preoperatively to 54/17 mm Hg postoperatively. During admission to ICU, patient experienced decreased Spo2 and Pao2 and respiratory acidosis. Chest X-Ray revealed pulmonary edema. Volume View^TM^/EV1000 was used for help to diagnosis of the cause of pulmonary edema. Increased levels of Extravascular Lung Water Index (EVLWI) and Pulmonary Vascular Permeability Index (PVPI) were consistent with acute respiratory distress syndrome (ARDS) due to reperfusion injury. The treatment included: adjustment of mechanical ventilation according to ARDS (ACV mode: Fio2 = 100%, TV = 500cc, PS = 12 cmH2O and PEEP = 5 cmH2O which increased to 12 gradually), negative fluid balance and administration of furosemide and deep sedation. The patient’s inotropes were changed to dobutamine and norepinephrine. With continuation of therapeutic interventions, the patient’s clinical and hemodynamic conditions improved, and finally, with normalization of EVLWI and PVPI, patient was extubated on fifth day. The post-operative echocardiography showed an ejection fraction of 55%, moderate right ventricular dysfunction, a mean systolic pulmonary pressure of 45mm Hg, and no remarkable sign of thrombosis in the main pulmonary artery and its right and left branches.

 The patient was discharged from the hospital on the 11th day after the operation in good general condition. Further information regarding his monitoring during his admission to ICU is available in Table 2.

**Table 2:** Volume View^TM^/EV1000^TM^ (Transpulmonary Thermodilution)

	**ELWI** **(ml/kg)**	**PVPI **	**Cardiac index** **(L/min/m**^2^**)**	**Cardiac output** **(L/min)**
2^nd^ day in ICU	12.9	3.1	3.4	6.8
3^rd^ day in ICU	9.8	2.2	3.5	6.9
4^th^ day in ICU	11.1	2.7	3.3	6.3
5^th^ day in ICU	9.4	1.8	3.1	5.6

###  Case 2

 A 52-year-old man with severe three-vessel disease was a candidate for Coronary Artery Bypass Graft (CABG) surgery. The pre-operative echocardiography showed left ventricular ejection fraction of 10 to 15%, severe left ventricular enlargement, and mild to moderate mitral regurgitation. Along with routine hemodynamic monitoring for cardiovascular surgery and after induction of anesthesia, advanced hemodynamic monitoring started using FloTrac sensor and Central Venous Oximetry Catheter (Scvo2) with Vigileo platform (Edwards Lifesciences, Irvine, CA, USA) ^12,13,14^. The stroke volume, cardiac index and Scvo2 were 46cc, 1/6 L/min/m2 and 73% after induction, respectively. Transesophageal echocardiography (TEE) showed a stroke volume of 50.1 cc, cardiac output of 2.97 L/min, and cardiac index of 1.63 L/min/m^2^ (acceptable agreement between the findings of both monitoring). After initiating the surgery and due to sympathetic stimulation of the heart, cardiac index (2.7 L/min.m^2^), Scvo2 (87%), blood pressure (114/65 mm Hg), pulse rate (73 per min), and central venous pressure (9 mm Hg) increased. The surgery was performed uneventfully, and the patient separated from CPB with epinephrine support(0.1mcg/kg/min). Postoperative TEE revealed the ejection fraction increased up to 35% and left ventricular outflow tract velocity time integral (LVOT VTI) also increased from a preoperative value of 14 to 26.3, postoperatively.

 The patient was transferred to ICU with increased epinephrine infusion from 0.03µg/kg/min to 0.05µg/kg/min and increased nitroglycerin infusion. The patient was weaned from mechanical ventilation and extubated in stable condition. During three days in ICU, cardiac index and Scvo2 increased from 2 L/min/m2 and 68% on arrival in ICU, to 3.3 L/min/m2 and 74% on the third day, respectively.

###  Case 3

 A 2-month-old female infant (4600 gr weight) was a candidate for a complete repair of type I truncus arteriosus. After induction of anesthesia with midazolam, fentanyl, and cisatracurium and routine monitoring for cardiac surgery, an oximetry catheter PediaSat (Edwards Lifesciences, Irvine, CA, USA) for continuous Scvo2 monitoring was inserted via right internal jugular vein. First hemodynamic variables were HR = 140/min, IBP = 95/36 mm Hg, CVP = 5mm Hg, SPo2 = 98% and Scvo2 = 74%. At the end of surgical repair, due to severe pulmonary hypertension and to prevent right ventricular failure, an ASD (Atrial Septal Defect) was created by the surgeon. The patient was weaned from the cardiopulmonary bypass (CPB) with the support of milrinone(0.5mcg/kg/min). Ten minutes after separation from CPB, Scvo2 dropped to 57%. With considering Pao2, hemoglobin and cardiac output as affecting factors in Scvo2 and with acceptable result of blood gas analysis and hemoglobin (Hb = 10.4 g/dl), low cardiac output was considered as reason for dropped Scvo2.Thus, epinephrine (0/05mcg/kg/min) was started and with a gradual increase in heart rate and blood pressure, Scvo2 reached 74% within 15 minutes. The patient was transferred to the ICU with Scvo2 of 74%, blood pressure of 87/49 mm Hg, pulse rate of 153/min, and oxygen saturation of 96%. The Scvo2 remained stable during the next day (73%) and the patient extubated without any problem. Postoperative period was uneventful and finally, the patient discharged from the hospital 10 days later.

###  Case 4

 A 62-year-old man with a history of CABG three years ago was admitted for the correction of infrarenal aortic aneurism. The pre-operative echocardiography showed an ejection fraction of 50 to 55%, severe tricuspid regurgitation (TR), and mild to moderate aortic insufficiency (AI), abdominal ultrasonography indicated fusiform dilation of the infrarenal aorta with a size of 50 * 68 mm. The routine monitoring included ECG, pulse oximetry, IBP, CVP, capnography and BIS (Bispectral index) were applied. Induction of anesthesia began with administration of midazolam, sufentanil and rocuronium. Aortic cross clamp and de-clamp in this type of surgery cause noticeable hemodynamic changes, thus monitoring the changes during the operation and applying therapeutic interventions at the right time are necessary. Therefore, we decided to use Flotrac sensor for our patient to help to follow the hemodynamic status during aortic clamping and after de-clamping of aorta. FloTrac (Edwards Lifesciences, Irvine, CA, USA) system provides cardiac output (CO), cardiac index (CI), stroke volume (SV), stroke volume variation (SVV), systemic vascular resistance (SVR). Considering aortic cross clamping leads increase in catecholamines, afterload, preload and blood pressure above the clamp, we monitored these hemodynamic variables using FloTrac system and applied suitable drug (labetalol, sodium nitroprusside, inhalational anesthetics and TNG). Aortic cross clamp took about 120 min and after completing surgical repair and acceptable hemostasis, the patient was ready to aortic de-clamping. Aortic de-clamping causes decrease in blood pressure, myocardial contractility, central venous pressure and cardiac output. Therefore, we carefully monitored these hemodynamic variables and performed necessary interventions, using blood transfusion, intravascular fluids, epinephrine, phenylephrine and correction of acid-base derangements. The patient transferred to ICU with an epinephrine infusion of 0.1 mcg/kg/min. In second postoperative day, according to adequate awakening, normal range measured hemodynamic variables by FloTrac system, sufficient urinary output and no acid-base disturbance, the patient was separated from mechanical ventilation. The patient was then transferred to the surgical ward after four days in stable condition. Further details regarding the patient’s hemodynamic condition are summarized in Table 3.

**Table 3:** The variation of the hemodynamic parameters before, during, and after clamping and de-clamping of the aorta (case 4)

**Time**	**Cardiac index** **(L/min/m**^2^**)**	**Central venous pressure** **(mm Hg)**	**Cardiac output** **(L/min)**	**Mean arterial pressure** **(mm Hg)**	**SVR** **(dynes-sec/cm–5)**	**SVRI** **(dynes-sec/cm–5/m2)**
7:19 a.m.	1.5	3	2.7	60	1689	2550
8:44 a.m.	2.5	5	3.9	89	1723	2602
8:48^1^ a.m.	3.4	5	5.2	120	1769	2672
8.53 a.m.	1.7	4	2.6	63	18.5	2741
9:24 a.m.	1.8	2	2.8	56	1543	2330
9:58 a.m.	2	3	3	69	1760	2658
10:39^2^ a.m.	2.1	3	3.3	71	1648	2489
11:14 a.m.	3.5	4	5.4	104	1481	2237
11:49^3^ a.m.	2.6	2	3.8	78	1600	2416
11:53^4^ a.m.	2.9	3	4.1	66	1229	1856
11:59^5^ a.m.	3.9	4	5.8	84	1103	1666
13:37^6^ a.m.	2.4	9	3.8	78	1453	2193
13:57^7^ a.m.	2.8	0	4.5	100	1778	2684
14:47^8^ a.m.	2.8	5	4	96	1820	2748
16:40 a.m.	3.6	10	5.4	81	1052	1588
7:53 a.m. (next day)	3	4	4.8	105	1683	2542

1: right after aortic clamping; 2: 2 hours after aortic clamping; 3: before first de-clamping attempt; 4: during de-clamping; 5: re-clamping the aorta; 6: before second de-clamping attempt; 7: right before the second de-clamping attempt; 8: completed de-clamping of the aorta.

###  Case 5

 A 75-year-old man with dilated cardiomyopathy was a candidate for inserting Left Ventricular Assist Device (LVAD) type Heartmate III (Abbott Laboratories, Abbott Park, IL, USA). The preoperative echocardiography indicated LVEF = 5-10%, severe right ventricular dysfunction and moderate TR. Routine monitoring for these patients are the same as other cardiac surgeries including ECG, IBP, CVP, BIS, cerebral oximetry, pulse oximetry and capnography. In these patients, due to the change in blood flow after LVAD implantation, sensors like Flotrac which use arterial pressure waveform analysis to calculate hemodynamic variables such as stroke volume and cardiac output, cannot be use. Moreover, we are exposed to Right Ventricular (RV) failure for several reasons after LVAD implantation. Therefore, insertion of PA catheter or Swan Ganz catheter is necessary in these patients. For our patient we used Swan Ganz CCOmbo catheter (Edwards Lifesciences, Irvine, CA, USA) to benefit from measuring mixed venous oxygen saturation (Svo2) as a general estimation of tissue perfusion in addition to pulmonary artery pressure monitoring. The arterial line was inserted in left femoral artery. Induction of anesthesia performed with sufentanil, etomidate and rocuronium. Initial hemodynamic parameters were: IBP = 119/83 mm Hg, PAP = 65/25 mm Hg, HR = 75/min. Spo2 = 100% and Svo2 = 75%.

 The surgery was performed without any particular problem and the patient was wean from cardiopulmonary pump with support of epinephrine (0.2 mcg/kg/min), vasopressin (2u/h) and milrinone (0.5 mcg/kg/min). Svo2 was 80% during CPB, but 10 min after weaning from CPB, Svo2 declined to 57%. Considering acceptable IBP and PAP and suitable intraoperative echocardiographic findings, we suspected low hemoglobin (7.3 g/dl) as the cause of Svo2 drop. Thus, transfusion of 1unit packed cell increased Svo2 to 72%. At the end of procedure, due to increasing dose of inotropes and vasopressors and lack of complete control of bleeding, IABP inserted and the patient was transferred to ICU with open chest. On second postoperative day, with the improvement of hemodynamics status and bleeding control, the sternum was rewired. During postoperative period, Svo2 used as one of the most valuable criteria for hemodynamics evaluation. Finally, with stabilization of hemodynamics, removal of IABP and significant reduction in dose of inotropes, the patient was weaned from mechanical ventilation and was extubated on 5th postoperative day and transferred to ward on 11th day.

## Discussion

 Since the last decade, advanced hemodynamic monitoring has been an essential tool which provides real-time information about cardiovascular systems and GDT utilizes this information to optimize perioperative management in Cardiac surgery.^15,16,17^ These strategies aim to optimize hemodynamic stability and guide interventions to improve patient outcomes. These technologies are expanding, and choosing the best advanced hemodynamic monitoring for a patient involves considering several factors. The goal is to select the technology that provides the most accurate and relevant hemodynamic data while considering the patient’s safety, comfort, and overall care. Here, we reported our experience over five various cases in a heart center hospital to show how they can improve decision-making. However, it took some time for the operating room and ICU staff to get trained in these technologies.

 The advanced hemodynamic monitoring based on arterial pressure waveforms (APW) such as FloTrac are uncalibrated and measure CO continuously. They have become a part of routine practice in the operating room and ICU because they are easy to handle, minimally invasive, and can provide information for the GDT strategy.^18,19^ While calibrated APW technologies like VolumeView are more accurate in the measurement of SV or CO and for tracking the changes in CO. Both calibrated and non-calibrated arterial pressure waveform monitoring, some of them are listed in Table 1, have their own advantages and limitations. The choice between them depends on the specific clinical context, the available resources, and the goals of hemodynamic management for each individual patient.

**Figure 1:**
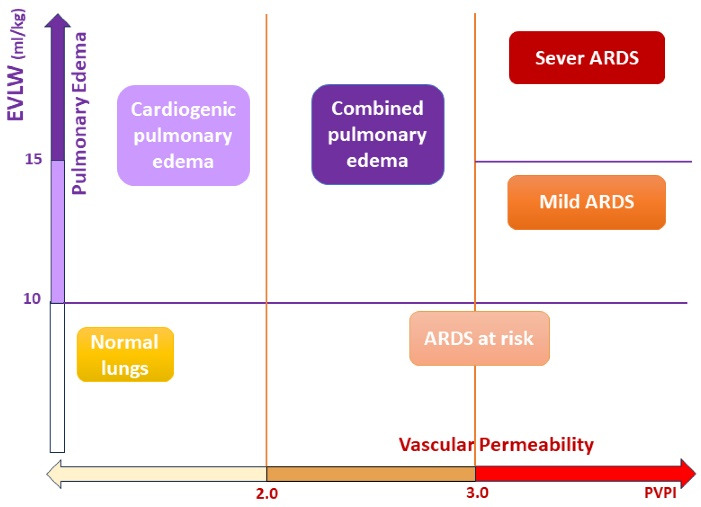
Extravascular lung water and vascular permeability diagram

 Reperfusion lung injury is the most common early complication after pulmonary endarterectomy, which occurs in about 9-10% of cases and manifests with hypoxia ^20,21^ In case 1, after excluding other causes of hypoxia, we used VolumeView/EV1000 platform to confirm the clinical suspicion. The increase of EVLW and PVPI, respectively, indicate the increase of extravascular fluid and vascular permeability in the lung, which confirms the diagnosis of ARDS caused by pulmonary microvascular damage, depicted in Figure 1. After confirming the diagnosis, treatment was started, and at this stage, periodic measurements of VolumeView parameters (EVLW and PVPI) were helpful in evaluating the response to treatment and determining the appropriate time to weaning from mechanical ventilation and tracheal extubating. If there was no acceptable response to the treatment and the intensity of hypoxia increased, ECMO could be considered.

 One big area in cardiac anesthesia is the patients with low ejection fraction^22^ who undergo CABG, and the anesthetic management of these patients can be complicated in some critical situations, such as induction of anesthesia, weaning from CPB, and post-operative care in ICU. Continuous advanced hemodynamic monitoring is crucial for decision-making on these patients as we need to have precise control of the cardiac output and the factors affecting it, which are: stroke volume, cardiac contractility, heart rate, preload, and afterload. We had a similar situation in case 2, in which we managed using FloTrac and Scvo2 monitoring.

 In aortic surgeries that require aortic cross-clamping, like case 4 here, we see extensive hemodynamic changes after aortic clamping and after releasing the cross-clamp. With aortic cross-clamp, we usually witness these hemodynamic changes: hypertension above the cross-clamp and hypotension below the cross-clamp, increase in CVP, increase in segmental wall motion abnormalities, metabolic changes including decrease in total oxygen consumption, decrease in CO2 production, increase in Svo2, metabolic acidosis and respiratory alkalosis. By releasing the cross-clamp, we will have a hypotension, decrease in CVP and cardiac output. Since inserting the arterial line to measure invasive blood pressure in cardiovascular surgery is necessary, FloTrac can be easily used in these patients.^23^ Stroke volume can be measured by the area under the arterial pressure waveform, and accordingly, the cardiac output and cardiac index are obtained.^24^ The SVV, if the patient is under positive pressure ventilation, is considered as a measure of intravascular volume and preload. If the CVP value is recorded, SVR is calculated as a measure of afterload. The additional information derived from advanced hemodynamic monitoring complements conventional monitoring, resulting in better patient management. This fact agreed with our observations for fluid therapy, inotrope, vasopressor, and vasodilator selection in cases 2 and 4.

 In pediatric cardiac surgery, we have a limited range of technology in advanced hemodynamic monitoring. Conventional monitoring like IBP and CVP are not enough in complex pediatric cardiac surgeries. On the other hand, the current sensors/catheters that are used in adult hemodynamic monitoring cannot be used for pediatrics due to their low weight. In these cases, Scvo2 monitoring can be a good option. Scvo2 is a balance indicator between oxygen supply and consumption. This balance is affected by the amount of blood hemoglobin, arterial oxygen saturation, and cardiac output. Therefore, when faced with a drop in Scvo2 along with other signs of hemodynamic instability, we should think of the correction of hemoglobin, oxygenation status, and parameters affecting cardiac output, which we experienced in case 3. It is important to note that in most cases, central venous oxygen saturation (Scvo2) is consistent with mixed venous oxygen saturation (Svo2), and of course, normal or high Scvo2 values can indicate cellular dysoxia.

 Left ventricular assist device (LVAD) is used as a bridge to transplantation or as destination therapy. After LVAD implantation due to change in pulsatility of the arterial pressure (the greater the contribution of LVAD to cardiac output, the lower pulsatility of flow) sensors like FloTrac which use arterial pressure waveform analysis to calculate hemodynamic variables, cannot be used. In addition, change in LV geometry plus increased venous return can cause right ventricular failure. Therefore, insertion of PA catheter or Swan Ganz catheter is necessary in these patients. In case 5, we used Swan Ganz CCOmbo catheter (Edwards Lifesciences, Irvine, CA, USA) to benefit from measuring mixed venous oxygen saturation (Svo2) as a general estimation of tissue perfusion in addition to pulmonary artery pressure monitoring.

## Conclusion

 In summary, the integration of advanced hemodynamic monitoring into patient management in cardiac surgery has the potential to revolutionize care delivery. By providing accurate and real-time information, these monitoring techniques enable proactive interventions, improved fluid management, early detection of complications, and individualized patient care. Incorporating such monitoring methods into routine practice has the potential to enhance patient outcomes, reduce morbidity, and contribute to the advancement of cardiac surgery.

## Aknowladgments

 We specially thank the Cardiac Anesthesiology Department,Rajaie Cardiovascular Medical and Research Center,for writing this article.

## Competing Interests

 The authers declare to conflict of interest in this study.

## Ethical Approval

 This study was approved by Research Ethics Committees of Rajaie Cardiovascular Medical and Research Center (Code: IR.RHC.REC.1402.050).

## Funding

 This research received no financial support.
